# Research and Remedies

**Published:** 1912-12-21

**Authors:** 


### RESEARCH AND REMEDIES.

### The Inunction Treatment of Measles.

The eucalyptus treatment of scarlet fever, advocated so strenuously by Dr. Milne, has already attracted a good deal of attention in the medical profession and among the laity. The general opinion seems to be that the system does not achieve all that Dr. Milne claims for it, and has been to some extent a disappointment. Dr. Connolly has lately tried a similar line of practice for measles in the Manchester Union Hospital, Crumpsall; his observations extend over 160 cases drawn from the poorest and least sanitary quarters of Manchester. The results are set forth in tabular form in the *Practitioner*, and the conclusion which the author draws is that a decided value attaches to the eucalyptus oil treatment of measles, combined with antiseptic treatment of the mouth and throat and first-rate nursing. The maintenance in the wards or rooms occupied by the patients of a uniform temperature of 65° F. is also laid stress on. Particulars of the whole series of cases are given, and it is certainly "up to" fever hospital officers to try this system and report their experiences.

### Antidote for Carbolic Acid Poisoning.

In the *South African Medical Record* Mr. J. Maberly publishes a case in support of his contention that the best antidote for carbolic acid is tincture of iodine; he has previously published three cases in the *Lancet* about five years ago. In the present case he was called to a child of eighteen months which had swallowed, twenty minutes previously, a tablespoonful (estimated) of pure Jeyes' fluid, and was in great pain. The author mixed half a drachm of tincture of iodine with half a wineglassful of water; after swallowing this the child got speedy relief. He gave instructions that a similar quantity was to be given after an hour, and next day found the child quite recovered. He holds that iodine and carbolic acid are mutually deprived of their virulence by admixture in equal quantities, and suggests that carbolic may prove of equal value as an antidote to iodine poisoning. If this thesis is correct it is difficult to see any virtue in the old-fashioned practice of using iodised phenol as a corrosive for the uterine mucosa; and, indeed, this substance seems to have fallen more or less into disuse.

### A Cure for Opium Eating.

Some time ago a plant called *Combretum sundaicum* was much lauded as a cure for the opium and morphine habits. The claims made on behalf of this drug were noted in The Hospital at the time, but not very much has been heard lately of its virtues. In the *Indian Medical Gazette* Mr. W. C. Brookes gives his experience with the crude drug and with a preparation made from it named Antipav. In thirty cases treated with decoction of combretum there was complete success in 90 per cent.: the patients were Chinese opium-eaters. With Antipav he has had less experience, but is able to record five successful cases. The method of using combretum is as follows: An ounce and a-half of the small twigs and leaves are placed in water in a large covered vessel, and are then simmered for four hours and the liquid strained. Then the decoction is further simmered without any cover on the vessel until its bulk is a quart. This is divided equally between two bottles A and B. In the bottle A is also placed the quantity of opium which the patient is accustomed to swallow in twenty-four hours, and the mixture well shaken. The patient then swallows an ounce of the contents of A, and the bottle is refilled from bottle B. This process continues eight times a day until bottles A and B are exhausted. The effect, of course, is to maintain the dose of combretum while diminishing that of opium. It is said that by this means the taste for opium is rapidly lost and the patient freed from the craving. It may be doubted whether the Western morphia-taker would respond as readily as the Eastern opium-eater to this treatment.

### Vicarious Menstruation.

This curious condition was at one time regarded with a certain amount of scepticism by some gynæcologists. No doubt some of the reported cases were capable of other explanations; but others stood the most stringent investigation successfully. An interesting case in point is recorded in the *Journal of Obstetrics and Gynæcology of the British Empire* by Craig and Kynoch. A child of eight possessed a suppurating gland which discharged through a sinus on the cheek. It was curetted, and afterwards closed; but at intervals it tended to break down again. At fourteen she had a small pin-point opening at the upper end of the scar left by the operation; and through this at intervals she lost blood. Uterine menstruation meanwhile was not yet established. Fifteeen months afterwards the sinus was soundly healed. It had not bled for four months; but at four equal intervals there had been a discharge of blood from the inner canthus of the left eye. This went on with great regularity every month for a further six months. The normal menstrual flow then became established for the first time; and from that time on there was never any hæmorrhage from either of the vicarious sources.

